# Analyzing the Effectiveness of the Push-Up Method for Idiopathic Scoliosis: A Preliminary Report

**DOI:** 10.7759/cureus.106463

**Published:** 2026-04-05

**Authors:** Hiroshi Kuroki, Naoya Tajima, Takuya Nagai, Hideaki Hamanaka, Naosuke Kamei

**Affiliations:** 1 Department of Orthopaedic Surgery, National Hospital Organization Miyazaki Higashi Hospital, Miyazaki, JPN; 2 Department of Orthopaedic Surgery, Nozaki Higashi Hospital, Miyazaki, JPN; 3 Department of Orthopaedic Surgery, University of Miyazaki Faculty of Medicine, Miyazaki, JPN

**Keywords:** conservative treatment, idiopathic scoliosis, physiotherapy scoliosis-specific exercises (psse), preliminary study, push-up method

## Abstract

Background and objective

As the standard of care for idiopathic scoliosis, observation is indicated for patients with curves between 10° and 25° who are still growing. However, if any treatment capable of controlling curve progression were offered at such an early stage, it might reduce the number of patients requiring orthotic therapy and, ultimately, surgery. We have devised the push-up method, which focuses on the gravity-based traction force for scoliosis deformity. In this study, both the temporary, immediate curve-correcting ability and the clinical outcomes of the push-up method in patients with idiopathic scoliosis were investigated to explore the potential of this procedure as a conservative treatment.

Methods

When performing the push-up method, the patient first sits on a high stool equipped with specially attached armrests and then lifts their buttocks by extending their elbows on the armrests. While maintaining this position, a continuous extension force is applied to the trunk to correct the scoliotic curve. Patients were instructed to perform 10 push-ups of 20 seconds each, followed by 40 seconds of rest, twice daily in the morning and evening. First, the changes in Cobb angles before and during the push-up position were evaluated in 20 curves from 13 patients with idiopathic scoliosis (all female) with a mean age of 15 years. Furthermore, changes in back muscle activity caused by the push-up position were assessed using electromyography. Next, Cobb angles before treatment and more than one year post-treatment were compared in 21 curves from 12 patients with idiopathic scoliosis (11 female, 1 male) with a mean age of 15 years.

Results

The average Cobb angle of 20 curves significantly decreased by approximately 7.4° during the push-up position, from 22.0 ± 2.6° to 14.6 ± 4.7°, yielding an average correction rate of 33.6%. Electromyography evaluation showed that the push-up position significantly increased activity in the latissimus dorsi muscles, while erector spinae muscle activity exhibited only mild changes. The main curve improved in 10 of the 12 patients (17 curves) and worsened in two patients (four curves). The average Cobb angle before treatment was 22.4 ± 2.9°, and at the final examination, it was 19.2 ± 6.1°, corresponding to an average correction rate of 14.3%.

Conclusions

Among the various procedures that use corrective traction forces for scoliosis, the push-up method is the first to be reported as specifically intended for therapeutic application. Based on the current preliminary investigation, we suggest that the push-up method could be considered as a conservative treatment option for certain patients with mild idiopathic scoliosis.

## Introduction

Idiopathic scoliosis is a structural, complex, three-dimensional developmental spinal deformity characterized by a coronal curvature of the spine exceeding 10°, accompanied by vertebral rotation [1,2,3]. If left untreated, idiopathic scoliosis may progress, and severe cases carry an increased risk of various morbidities and mortality [4]. In treating idiopathic scoliosis, the therapeutic approach is generally determined by both the Cobb angle and skeletal maturity. If the patient is still growing and the curvature measures between 20° and 40°, nonoperative treatments are recommended, whereas if growth is complete and the curvature is 40-50° or greater, corrective fixation surgery is indicated. However, for mild cases with a Cobb angle of 10° to 20°, no active treatment is currently available, and patients are only observed over time [5]. If interventions could be applied at this early stage to prevent or slow curve progression, it might be possible to reduce the number of patients who ultimately require orthotic therapy or surgery.

Corrective forces for scoliosis include traction on the concave side, compression on the convex side, pressure from both sides (three-point correction), and bending toward the convex side [6]. Of these, traction applied to the trunk is the easiest to implement. Axial traction for correcting spinal deformities is a concept with ancient origins, with devices such as Hippocrates’ ladder being used long before the Common Era [7]. However, mechanical methods for correcting spinal deformities gradually fell out of favor because they frequently caused paraplegia [7]. In 2016, Tajima et al. developed the push-up method, which focuses on gravity-based traction for scoliosis, and were the first to apply it clinically [8]. The purpose of this study was to evaluate both the temporary, immediate corrective effect of the push-up method on spinal curves and its therapeutic efficacy for patients with mild idiopathic scoliosis.

## Materials and methods

Push-up method

**Figure 1:**
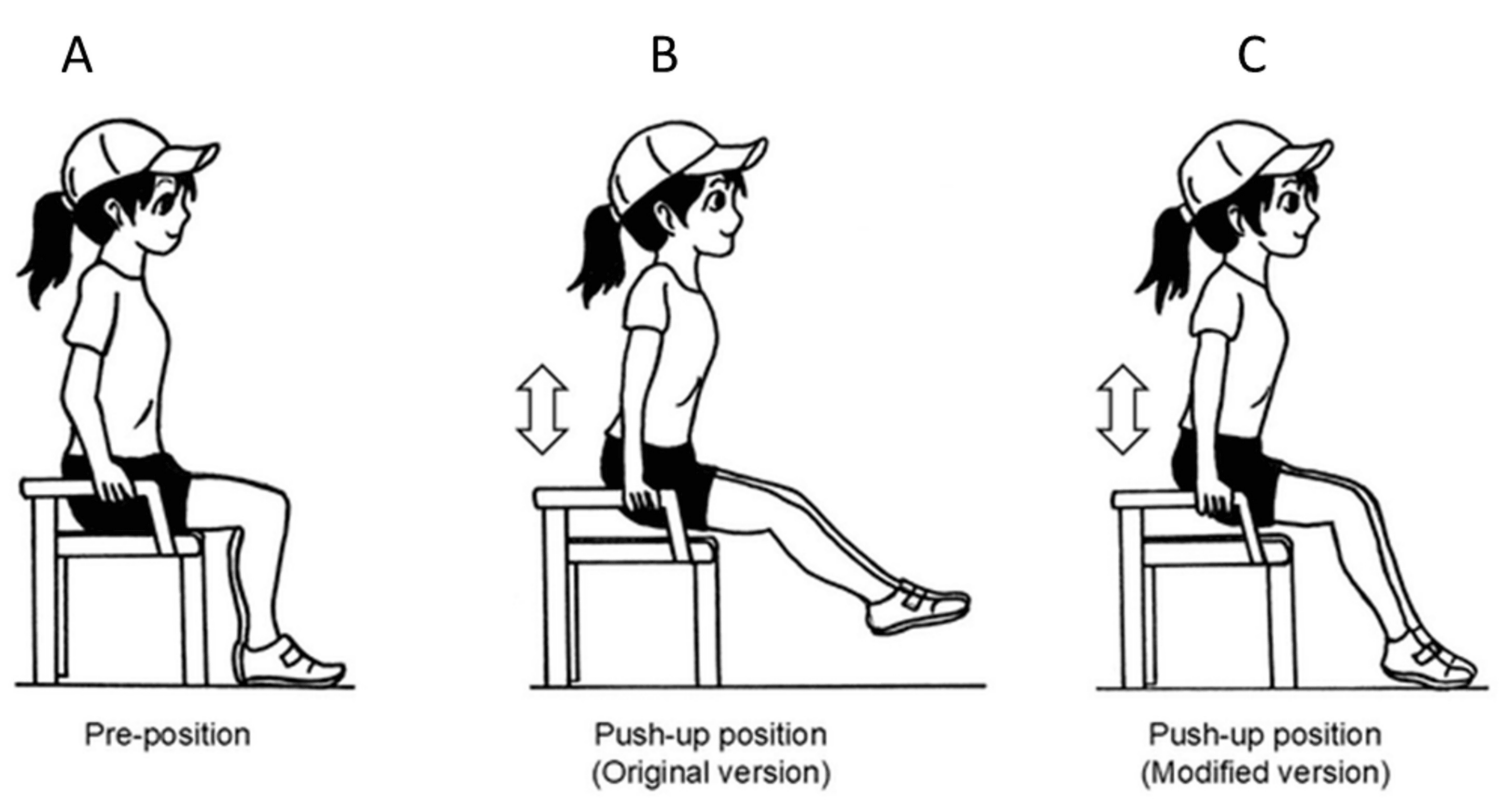
The push-up method A. Pre-position. B. Push-up position (original version). C. Push-up position (modified version) When achieving the push-up position, the patient first sits on the high stool that has specially attached armrests and then lifts their buttocks by extending the elbow joints on the armrests. Initially, they were instructed to completely lift the feet off the floor, but if their physical strain was too great to keep this position, the forefeet were allowed to contact the floor lightly Illustration by Yusuke Nomoto

To perform the push-up method, we designed a special chair with long legs, a seat height of approximately 75 cm, no backrest, and reinforced armrests. During the push-up movement, patients grasped the left and right armrests with both hands, then extended their arms and lifted their trunks off the seat, using their body weight to provide the traction force, and maintained this position for about 20 seconds. Initially, patients were instructed to lift their feet completely off the floor; however, if this caused excessive physical strain, the forefeet were allowed to lightly touch the floor (Figure 1).

Research 1

We investigated changes in Cobb angles before and during the push-up position in 13 patients with 20 curves of idiopathic scoliosis. All patients were female, with ages ranging from 12 to 18 years and a mean age of 15 years. The curve patterns included four cases of a single thoracic curve, one case of a single thoracolumbar curve, one case of a single lumbar curve, and seven cases of double curves (Table 1).

**Table 1:** Patient demographics - research 1 AIS: adolescent idiopathic scoliosis

Demographics - research 1	
AIS patients	13 cases (20 curves)
Gender	Male: 0 case/female: 13 cases
Age	Mean: 15 years (range: 12-18 years)
Curve type	Single thoracic: 4 cases/single thoracolumbar: 1 case/single lumbar: 1 case/double: 7 cases

Regarding the method, a plain frontal X-ray was first taken with the patient sitting in the chair, followed by another frontal X-ray after the patient maintained the push-up position for approximately 20 seconds. The Cobb angles from both images were then compared. We also used electromyography to assess changes in back muscle activity induced by the push-up position. Electrodes were placed bilaterally on the latissimus dorsi at the T7 level and on the erector spinae at the L4 level, and measurements were recorded with and without the push-up position. Muscle activity was evaluated using both muscle voltage (µV) and integrated electromyography (µVs).

Research 2

The clinical courses of 12 patients with idiopathic scoliosis (21 curves) were evaluated after undergoing push-up exercise therapy for more than one year (mean: 20 months, range: 12-24 months). There were 11 female and one male patient, with ages ranging from 12 to 18 years and a mean age of 15 years. The curve patterns included one case of a single thoracic curve, two cases of a single thoracolumbar curve, and nine cases of double curves (Table 2).

**Table 2:** Patient demographics - research 2 AIS: adolescent idiopathic scoliosis

Demographics - research 2	
AIS patients	12 cases (21 curves)
Gender	Male: 1 case/female: 11 cases
Age	Mean: 15 years (range: 12-18 years)
Curve type	Single thoracic: 1 case/single thoracolumbar: 2 cases/double: 9 cases

One set of the push-up treatment consisted of approximately 20 seconds of push-ups followed by 40 seconds of rest. Patients were instructed to perform 10 repetitions of this training twice daily, in the morning and evening. The initial training was conducted in the hospital, after which patients continued the exercises at home. We then compared Cobb angles before and after the intervention.

Correction rates were calculated using the following formulas:

Correction rate (corrective effect in research 1) = 

$\frac{\text{Cobb angle before push-up} - \text{Cobb angle during push-up}}{\text{Cobb angle before push-up}} \times 100$

Correction rate (treatment effect in research 2) =

$\frac{\text{Cobb angle before intervention} - \text{Cobb angle after intervention}}{\text{Cobb angle before intervention}} \times 100$

Radiographs

The X-ray radiographs were acquired using SONIALVISION Safire 17 (Shimadzu Corporation, Kyoto, Japan). The electromyograph used was a bio-monitor ME6000 (NIHON MEDIX CO., LTD., Kashiwa, Chiba, Japan). Radiographic measurements were performed in a blinded manner by physiotherapists who were not involved in the study, using standard techniques.

Statistical analysis

A two-tailed paired t-test was used to evaluate statistical differences in Cobb angles between the compared data. Analyses were performed using Microsoft Excel 2016 (Microsoft Corporation, Redmond, WA). A p-value of < 0.05 was considered statistically significant.

Ethical considerations

We obtained written informed consent from all patients and their guardians for publication of this report and any accompanying images. Furthermore, this study was approved by the Ethics Committee of Nozaki Higashi Hospital (approval number: NH-2018001), and all procedures were conducted in accordance with the principles of the Declaration of Helsinki.

## Results

Research 1

**Figure 2:**
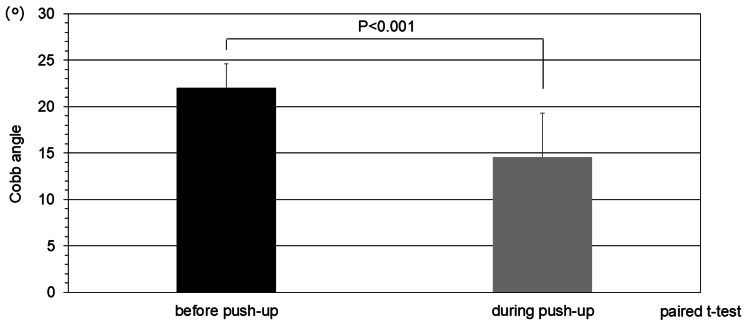
Corrective effect The average Cobb angle for all 20 curves was 22.0 ± 2.6° before push-up and 14.6 ± 4.7° during push-up, a significant decrease of approximately 7.4°, with an average correction rate of 33.6% (t-value: -6.26)

The average Cobb angle for all 20 curves was 22.0 ± 2.6° before push-up and 14.6 ± 4.7° during push-up, a significant decrease of approximately 7.4°, with an average correction rate of 33.6% (Figure 2).

**Figure 3:**
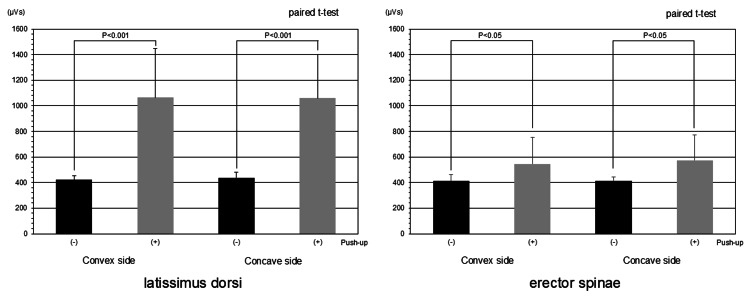
Electromyogram In the electromyographic evaluation, the push-up position significantly increased latissimus dorsi muscle activity, whereas erector spinae muscle activity showed only a mild change. The t-values of each were -6.04 for the convex side of the latissimus dorsi, -6.54 for the concave side of the latissimus dorsi, -2.19 for the convex side of the erector spinae, and -2.86 for the concave side of the erector spinae

In the electromyographic evaluation, the push-up position significantly increased latissimus dorsi muscle activity, whereas erector spinae muscle activity showed only a mild change (Figure 3).

Research 2

**Figure 4:**
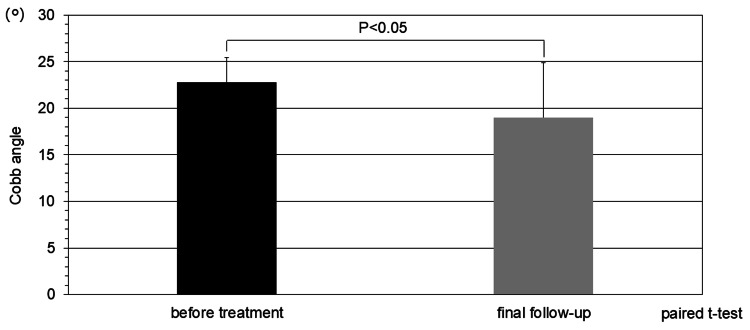
Clinical course The main curve improved in 10 of the 12 cases (17 curves) and worsened in two cases (four curves). The average Cobb angle was 22.4 ± 2.9° before treatment, but at the final examination, it was 19.2 ± 6.1°, a significant decrease of approximately 3.2°, and the average correction rate was 14.3% (t-value: -2.16)

The main curve improved in 10 of the 12 cases (17 curves) and worsened in two cases (four curves). The average Cobb angle was 22.4 ± 2.9° before treatment, but at the final examination, it was 19.2 ± 6.1°, a significant decrease of approximately 3.4°, and the average correction rate was 14.3% (Figure 4).

Illustrative cases

Effective Case: 16-Year-Old Girl With Double Thoracic and Lumbar Curves

**Figure 5:**
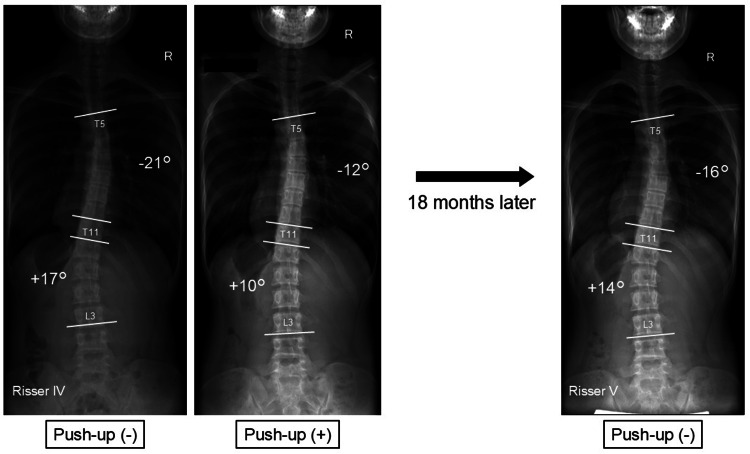
Effective case The patient had scoliosis of 21° right convexity in the thoracic spine and 17° left convexity in the lumbar spine, which was corrected to 12° and 10°, respectively, by the push-up method. After 18 months of performing the push-up method, the curvature had improved to 16° and 14°, respectively

The patient had scoliosis of 21° right convexity in the thoracic spine and 17° left convexity in the lumbar spine, which was corrected to 12° and 10°, respectively, by push-up position. After 18 months of performing the push-up method, the curvature had improved to 16° and 14°, respectively (Figure 5).

Ineffective Case: 13-Year-Old Girl With Double Thoracic and Lumbar Curves

**Figure 6:**
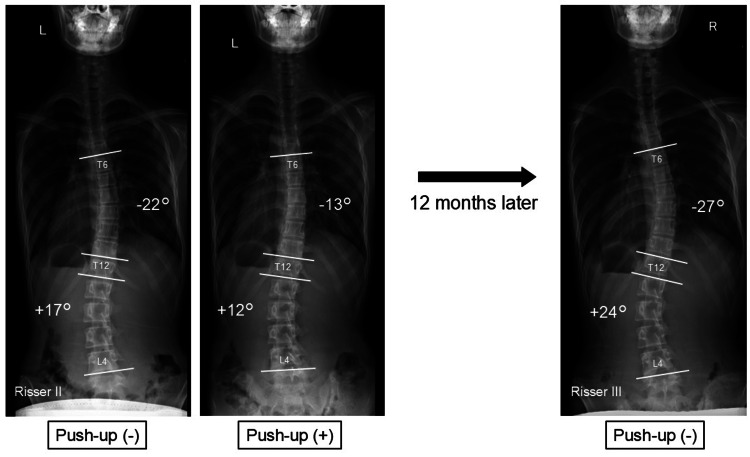
Ineffective case The patient had scoliosis of 22° right convexity in the thoracic spine and 17° left convexity in the lumbar spine, which was corrected to 13° and 12°, respectively, by push-up position. However, despite performing the push-up method for 12 months, the curvatures had progressed to 27° and 24°, respectively

The patient had scoliosis of 22° right convexity in the thoracic spine and 17° left convexity in the lumbar spine, which was corrected to 13° and 12°, respectively, by push-up position. However, despite performing the push-up method for 12 months, the curvatures had progressed to 27° and 24°, respectively (Figure 6).

## Discussion

Only observation, which is essentially equivalent to neglect, has so far been chosen as the standard approach for the treatment of mild idiopathic scoliosis with a Cobb angle of 25° or less. Nevertheless, most cases of scoliosis identified through screening fall into this range, making some form of intervention desirable. In fact, in clinical practice, we frequently hear sincere concerns from patients and their guardians, such as, "Is there anything we can do to prevent the scoliosis from getting worse?" However, we are currently unable to meet these expectations. At present, several physiotherapy scoliosis-specific exercises (PSSE) are being implemented mainly in Europe with the goals of improving scoliosis, preventing progression of the deformity, and supporting orthotic therapy [9]. However, their effectiveness has not yet been firmly established through strong scientific evidence, and therefore, they cannot yet be readily recommended.

In addition to push-ups, several other methods have been reported for the use of traction forces in scoliosis, including Cotrel [10], suspension [11], and hanging [12]. However, their purposes differ. Cotrel is primarily used to improve spinal flexibility before surgery, while suspension and hanging are primarily used to evaluate spinal flexibility before interventions such as surgery or orthotic treatment. In contrast, push-ups are specifically intended for therapeutic use. To the best of our knowledge, there have been no modern reports other than ours [8] of systematically applying gravity-based traction as a treatment for scoliosis within the available literature.

There are two types of pulling forces applied in exercise therapy for scoliosis: traction and stretching. Zakaria et al. [13] compared these two procedures and reported that stretching was more effective than traction in improving the Cobb angle. The Schroth method, a representative PSSE, utilizes multiple components such as auto-elongation, de-rotation, de-flexion, rotational breathing, stabilization, muscle strengthening, along with traction and stretching [14]. By contrast, the push-up method uses traction alone, so it may be less comprehensive than other PSSE approaches; however, the technique is simple and easy to teach and perform.

The mechanism underlying the therapeutic effects of the push-up method remains unclear. Electromyographic analysis of the back muscles in the push-up position showed increased activity of the latissimus dorsi, which is primarily involved in lifting the trunk, whereas changes in erector spinae activity were not significant. This suggests that adopting the push-up position may reduce trunk muscle tension, thereby facilitating spinal elongation, and it is hypothesized that traction generated by the patient’s own body weight contributes to the corrective effect. In the current study, the push-up method resulted in an average correction of 7.4° across all 20 curves, and improvement was observed in 10 of 12 cases following more than one year of continued practice. These results suggest that the push-up method may serve as a conservative treatment option for mild idiopathic scoliosis.

The advantages of the push-up method are as follows: it requires no specialized equipment other than a simple chair, is noninvasive, can be performed at home in a short time, and can be used in combination with orthotic therapy. On the other hand, the disadvantages are that it may not be feasible or effective for patients with weak upper limb strength, reduced trunk flexibility, a primary curvature in the upper thoracic spine, or severe and advanced scoliosis.

Limitations and future directions

Because this research is a preliminary investigation aimed at exploring practical application, several limitations should be acknowledged. These include a small sample size; clinical evaluation conducted before skeletal maturity in some cases; reliance on patient adherence for exercise performance, which limits accuracy; assessment based solely on the Cobb angle without incorporating appearance or patient-reported outcomes; inclusion of heterogeneous curve patterns; absence of a control group, preventing comparison with natural progression; and lack of multivariate adjustment for potential confounders. Furthermore, the observed changes in Cobb angle were small over time, making it difficult to rule out the influence of measurement error.

In the future, to strengthen the level of evidence, it will be necessary to increase the sample size and collect data using more rigorous study designs to clarify the mechanism of action, identify factors distinguishing effective from ineffective cases, determine optimal treatment protocols, and evaluate potential synergistic effects when combined with orthotic therapy.

## Conclusions

As an exercise therapy for idiopathic scoliosis, we devised the push-up method, which applies the gravity-based traction force to the trunk, and verified its effectiveness. The push-up method resulted in an average correction of 7.4° for all curves, with improvement observed in most cases after more than one year of continued practice. Although it is necessary to increase the sample size and gather additional data, these findings suggest that the push-up method, alone or combined with orthotic therapy, may serve as a supportive conservative treatment for mild idiopathic scoliosis.
